# Hospital Meetings

**Published:** 1906-02-10

**Authors:** 


### HOSPITAL MEETINGS.

### GENERAL LYING-IN HOSPITAL.

The annual meeting of the General Lying-in Hospital, York Road, Lambeth, was held at the hospital on January 31. The chair was taken by Sir John Williams.

The report for the past year shows that the alterations and additions to the hospital have now been practically completed by the erection of another story. These alterations had been urged upon the hospital by the representatives of King Edward's Hospital Fund, and secure proper accommodation for the nurses and servants, and also provide due safeguards against fire, in addition to five more beds for the hospital. The cost of these alterations is about £10,000, and this sum the Committee are now asking for; the work of the hospital will be much crippled until this amount is obtained. It is many years now since the hospital has made any appeal to the public for funds. Its finances have always been managed on business lines, legacies being placed to capital account and never being treated as income. No charge of hospital extravagance has ever been brought against the Committee, and they have worked on the principle that a charitable institution should, like a business establishment live within its income.

The medical report shows that 614 in-patients have been delivered during the year, of whom one died. The total is a record one, and fewer women have been refused admission this year than ever before; the Committee trust that when they have five more beds at their disposal they will not be compelled to turn many applicants away from the hospital. Two hundred and fifty-nine women were attended in their own homes. The arrangements for the training of nurses and midwives have suffered somewhat on account of the alterations to the hospital, but in spite of this their numbers show an increase, and good work in this direction has been accomplished during the past year.

The report and balance-sheet having been adopted, customary votes of thanks were passed, including one to the Chairman, moved by the Hon. A. de Tatton Egerton.

Sir John Williams, in responding, recalled the changes that had passed over the hospital since he had first been connected with it, and the rapidity with which its work had grown. For many years the hospital had enjoyed a great reputation for the training of nurses and of midwives, and they were glad to think that they had been able to assist in the successful carrying out of the Midwives Act.

